# Achieving Complete Remission of Hepatocellular Carcinoma: A Significant Predictor for Recurrence-Free Survival after Liver Transplantation

**DOI:** 10.1155/2019/5796074

**Published:** 2019-01-08

**Authors:** Christin Bürger, Miriam Maschmeier, Anna Hüsing-Kabar, Christian Wilms, Michael Köhler, Martina Schmidt, Hartmut H. Schmidt, Iyad Kabar

**Affiliations:** ^1^Department of Medicine B for Gastroenterology and Hepatology, University Hospital Muenster, 48149 Muenster, Germany; ^2^Department of Clinical Radiology, University Hospital Muenster, 48149 Muenster, Germany

## Abstract

**Background:**

Liver transplantation (LT) is a curative treatment for hepatocellular carcinoma (HCC) and the underlying primary liver disease; however, tumor recurrence is still a major issue. Therefore, the aim of this study was to assess predictors and risk factors for HCC recurrence after LT in patients within and outside the Milan criteria with a special focus on the impact of different bridging strategies.

**Methods:**

All patients who underwent LT for HCC between 07/2002 and 09/2016 at the University Hospital of Muenster were consecutively included in this retrospective study. Database research was performed and a multivariable regression analysis was conducted to explore potential risk factors for HCC recurrence.

**Results:**

A total of 82 patients were eligible for the statistical analysis. Independent of bridging strategy, achieving complete remission (CR) was significantly associated with a lower risk for tumor recurrence (*p = 0.029*; OR = 0.426, 95% CI 0.198-0.918). A maximal diameter of lesion < 3 cm was also associated with lower recurrence rates (*p = 0.040*; OR = 0.140, 95% CI 0.022-0.914). Vascular invasion proved to be an independent risk factor for HCC recurrence (*p = 0.004*; OR = 11.357, 95% CI 2.142-60.199).

**Conclusion:**

Achieving CR prior to LT results in a significant risk reduction of HCC recurrence after LT independent of the treatment modalities applied.

## 1. Introduction

Despite increasing approvals of novel antiviral drugs against hepatitis B and C, the incidence of hepatocellular carcinoma (HCC), the most common primary liver tumor, is still rising worldwide. Globally, it constitutes the 2^nd^ leading cause of cancer-related death [1–5]. Most HCC develop in a cirrhotic liver [6]. Alcoholic cirrhosis, active hepatitis B and C, and nonalcoholic fatty liver disease have been identified as the main underlying diseases. Prevalence is higher in males than in females. A more frequent exposition to risk factors is assumed to be one cause of this male predominance [7]. HCC is an often lethal disease with a combined 5-year survival rate of only about 15% in the USA and about 12% in Europe [8]. Liver transplantation (LT) is the favored treatment for patients with HCC and cirrhosis, as it can cure both the tumor with all intrahepatic foci and the underlying cirrhosis [5, 7]. HCC makes up about 20% of all indications for LT in Europe [8]. Waiting time for a deceased donor liver can be long due to shortage of cadaveric organs for transplantation. In many cases bridging strategies including surgery, loco-regional, molecular targeted, and radio-oncological procedures are applied aiming at prevention of tumor progression and subsequently gaining time to LT [9–12]. For patients with an advanced tumor stage, the same interventions are performed aiming at downstaging and thus making LT possible in the first place [13].

HCC recurrence is one significant problem after LT with recurrence rates of approximately 15-20%. Due to limited treatment options, prognosis of such recurrence is still poor with a median survival of less than 12 months after diagnosis [14]. Multiple possible risk factors have been investigated in order to optimize patient selection for transplant listing and to lower the risk of tumor recurrence post LT, but associated risk factors are still not completely evaluated. The Milan criteria (single lesion </= 5 cm or a maximum of three lesions </= 3 cm) are widely used as a decision basis for patient selection for LT, especially as HCC recurrence rates dropped significantly after application since 1996 [15]. However, using the Milan criteria as the only basis to select transplant candidates may result in excluding HCC patients who may still profit from LT. Several studies suggest alternatives, such as the University of California San Francisco (UCSF) criteria (single lesion <6.5 cm, maximum of three lesions with none >4.5 cm, and cumulative tumor size <8 cm) which render similar recurrence-free survival rates [16]. Biological features of the tumor (grading, microvascular or lymphatic invasion, alpha fetoprotein (AFP) level, and response to bridging therapies) also play an important role regarding HCC recurrence rates [17]. However, some of these features such as microvascular or lymphatic invasion cannot be assessed prior to LT. Tumor grading may be retrieved by liver biopsy, but there are some data indicating a higher risk of HCC recurrence by spreading tumor cells in the biopsy channel [18]. Posttransplant studies indicate that the immunosuppressive regimen may have an impact on tumor recurrence. Calcineurin inhibitors (CNI), the most widely used immunosuppressive medication, have been associated with increased tumor growth and a higher risk of tumor recurrence [19, 20]. Mammalian targets of rapamycin (mTOR)-inhibitors (Sirolimus, Everolimus) have antiproliferative and antiangionetic properties and some data indicate a protective role [21].

In the present study, we analyzed recipient- and donor-related predictors and risk factors for HCC recurrence after LT with a special focus on the role of different bridging modalities.

## 2. Materials and Methods

We conducted a single-center, retrospective study on patients who were treated for HCC and underwent LT between 07/2002 and 09/2016 at the University Hospital of Muenster and who received follow-up care at this center. Inclusion criteria were age over 18 years, HCC as the main indication for LT, available recipient, and donor data. All transplanted organs were retrieved from deceased donors and implanted in orthotopic technique. Extrahepatic tumor manifestations were ruled out immediately before transplantation by chest- and abdominal CT-scan and/or MRI. HCC recurrence was defined as any confirmed intra- or extrahepatic HCC lesion detected by radiographic or histopathological diagnostics after LT. Standard posttransplant follow-up included abdominal multislice-imaging (CT- or MRI scans) every 6 months and alpha-fetoprotein measurements as well as abdominal sonography in 3-monthly intervals; further diagnostics were conducted symptom oriented. Patient data were extracted from health care files at the University Hospital of Muenster. Approval to the study was given by the local ethical committee and it was conducted in conformity to the 1975 Declaration of Helsinki (7^th^ Revision of October 2013).

Demographic data collected for both recipients and donors were age, sex, and BMI. Further patient related demographic values were waiting time from HCC diagnosis to LT and survival time after LT. Furthermore, we evaluated the underlying hepatic diseases. Tumor related data and histopathological properties were gained from pre-LT radiological diagnostics and histopathological findings of pre-LT biopsy samples and the liver explants. Reported were maximum tumor size, the number of nodules, fulfilment of the Milan criteria, tumor grading (according to the Edmondson and Steiner grading system) [22], microvascular and lymphatic invasion, and the stage of liver fibrosis (according to the fibrosis score according to Batts and Ludwig) [23]. Clinical response to bridging therapies was subdivided into no detectable remission, partial or complete remission (CR) according to the level of tumor necrosis in the histopathological exam of the explant liver. The definition of CR was the absence of vital tumor in the explant liver. Partial remission was defined as presence of partial tumor necrosis but persisting vital tumor residues. Evaluated pre-LT bridging and downstaging treatments were transarterial chemoembolization (TACE), Sorafenib, surgical resection, selective internal radiation therapy (SIRT), or other radiation treatment and radiofrequency ablation (RFA). We also included the highest pre-LT AFP level, CRP-level directly before LT, and sampling of a pre-LT targeted biopsy of the tumor in this study.

### 2.1. Immunosuppression after LT

Post-LT-immunosuppressive regimen was documented. All patients received an intraoperative induction therapy with 500 mg of prednisolone. After LT, most patients received an immunosuppressive combination therapy. Maintenance immunosuppression comprised various combinations of the following three drugs: Tacrolimus and/or Everolimus and/or Mycophenolate Mofetil.

Statistical analyses were performed using IBM SPSS Statistics for Windows, version 24 (SPSS Inc., Chicago, Illinois).

## 3. Results

A total of 82 patients were eligible for the statistical analysis. The majority of LT recipients were male (82.9%) and mean age at LT was 57.2 ± 9.4. Mean donor age was 50.9 ± 15.4 years. The most common underlying liver diseases were alcoholic cirrhosis and viral hepatitis (B and C) in 25.6% and 51.2%, subsequently. The majority of patients had either liver cirrhosis (89%) or an advanced stage of liver fibrosis (4.9%, grade 3 according to Batts and Ludwig score) [23]. A targeted liver biopsy was obtained in 52 subjects (65.0%). Tumor grading ranged between well- and undifferentiated; 64.6% showed moderate differentiation (grade 2 according to the Edmondson and Steiner grading system) [22]. More than half of the included patients (53.7%) were outside the Milan criteria at time of HCC diagnosis. Median waiting time from HCC diagnosis until LT was 7 (IQR 2-12) months. 64 subjects received bridging treatment prior to LT. The majority of them underwent TACE (45, 54.9%). Remission could be reached in 59 subjects (71.9%). Of them, 43 (52.4%) achieved partial and 16 (19.5%) CR. Tumor recurrence after LT occurred in a total of 28 subjects (34.1%), and median recurrence-free survival time was 12.50 (IQR 6-28,25) months. A total of 36 patients died during the observed time period; 23 of them suffered from tumor recurrence. Median survival time of all patients after LT was 49.50 (IQR 24.50-84.75) months. Predictors for achieving a CR were the presence of only one tumor lesion (p=0.001; OR 10.7, 95% CI 2.7-42.0) and/or the fulfilment of the Milan criteria (p = 0.004; OR 7.1, 95% CI 1.8-27.4). AFP level (p=0.68) and the grade of liver fibrosis (p=0.37) were not statistically significant factors regarding achievement of CR. Further demographic and clinical data are depicted in Table 1.

Multivariable regression analysis revealed CR (*p = 0.029*; OR = 0.426, 95% CI 0.198-0.918), vascular invasion (*p = 0.004*; OR = 11.357, 95% CI 2.142-60.199), and maximal diameter of lesion < 3 (p = 0.040; OR = 0.140, 95% CI 0.022-0.914) to be statistically significant for HCC recurrence. Detailed results of univariable and multivariable analysis are included in Table 2.

**Figure 1:**
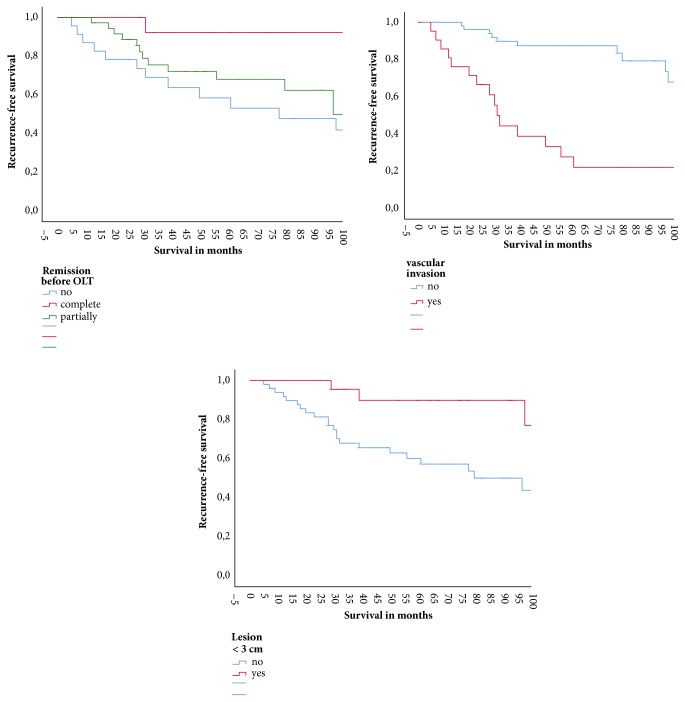


Kaplan-Meier curves in Figure 1 demonstrate the recurrence-free survival probability with regard to the different risk factors.

## 4. Discussion

HCC recurrence is a major issue after LT. Treatment options are limited and in the majority of cases, only palliative therapeutic measures are available [14, 24]. Further evaluation of risk factors and protective properties regarding tumor recurrence is necessary for assessing the hazard of each patient as well as in helping the treating physicians in developing new strategies to reduce HCC recurrence after LT. This is especially crucial in times of organ shortage.

The presence of only one lesion was the strongest predictor for achievement of CR prior to LT in our study, independent of its size. This finding supports data that aim to extend the selection criteria for transplant candidates such as the study by Yao et al. introducing the UCSF Criteria [16].

In our study, achieving CR prior to LT was significantly associated with reduced recurrence rates of HCC. This fact may be due to a less aggressive biological tumor behavior as discussed before [25–27]. Our data confirm the results of a former large scale study from the US Multicenter HCC Transplant Consortium (UMHTC) on patients inside the Milan criteria, which showed that achieving CR is crucial, leading to better outcomes in recurrence-free and overall survival [28]. However, our data suggest that achieving CR significantly reduces HCC recurrence rates after LT irrespective of whether patients were inside or outside the Milan criteria. This fact is consistent with some former data indicating that tumors outside the Milan criteria do not necessarily have less favorable biological properties and may thus respond to treatment modalities and benefit from LT likewise [29].

One further study of Yao et al. showed a significant reduction of HCC recurrence rates in patients in whom HCC downstaging into Milan criteria could be achieved using bridging procedures such as TACE and radio frequency ablation (RFA). Patients with CR prior LT showed a 5-year overall survival as well as a recurrence-free survival of > 90% while patients with no response to bridging treatment had a 5-year overall survival of only about 50% [13]. Besides locoregional therapies such as TACE and RFA, our analysis included surgical resection and combinations between surgery and locoregional antitumoral strategies in order to achieve CR. Interestingly, complete CR was able to significantly reduce HCC recurrence irrespective of the way and the number of interventions that were needed to achieve CR. Furthermore, in a study of Cucchetti et al., reaching CR in patients waiting for LT was able to lower dropout rates on the waiting list and showed resection to be one of the most effective treatment options [10]. From the oncological perspective, combination of resection and LT may be a favorable option. Additionally, the resected specimen renders valuable histological results on relevant biological properties such as grading and vascular invasion. However, applicability is often limited by impaired liver function and tumor multifocality.

In our study collective, recurrence rates were higher compared to other studies [14–16]. One reason could be that a considerable number of patients included were outside the Milan criteria. Of these, 31.7% had tumors larger than 5 cm in diameter.

One further major risk factor for HCC recurrence after LT in our study was vascular invasion. Whereas macrovascular invasion can be detected in preoperative diagnostics in most cases, microvascular invasion is regularly a postoperative finding. So far, no specific preoperative markers or radiological imaging techniques could be established to securely detect microvascular invasion in advance [30, 31]. Preoperative biopsy cannot always render conclusive results due to tumor heterogeneity leading to sampling errors [32, 33]. Generally, vascular invasion may indicate a systemic character of the HCC [34].

Another independent risk factor for HCC recurrence after LT in our study was an increasing tumor size. An increasing number of HCC lesions were correlated with a higher rate of HCC recurrence in the univariable regression analysis only. However, multivariable analysis showed no association between number of HCC lesions and HCC recurrence. This fact may be due to the extensive use of several bridging strategies at our center in order to achieve CR in each case irrespective of lesions number and tumor spread within the liver. These findings are partially consistent with studies that showed a favorable prognostic impact of the Milan criteria and lead to establish these for patient selection priority for LT [15]. However, our data indicate that patient selection for LT solely dependent on the Milan criteria seems to be too strict and may exclude patients that still greatly benefit from LT, especially as these criteria may discharge tumor biology and further significant prognostic factors such as tumor response to treatments prior to LT [16, 35–39].

Remarkably, in our study, performing targeted tumor biopsy before LT was not associated with increased HCC recurrence rates. This fact may debunk former results [18, 40, 41]. Therefore, performing a biopsy in HCC, especially in case of unclear liver masses as well as in cases with inconsistent contrast enhanced imaging, seems to be safe and even reasonable.

Time on the waiting list was not predictive for a higher risk for HCC recurrence in our study. This finding may be due to the fact that the majority of patients received bridging therapies and further underlines the major role of tumor biology on tumor progression [12, 42].

Data on recurrence protection using mTOR inhibitors is still controversial. Most study populations were small and until now, only few randomized controlled trials and prospective studies are available [21, 43, 44]. In our study, univariate analysis showed a strong tendency towards a significant reduction of HCC recurrence (*p = 0.053*) in patients who received an Everolimus based immunosuppression. However multivariable analysis showed no influence of Everolimus on HCC recurrence. Sirolimus based regimens were rare in our study collective and were thus not included in the statistical analysis. Nevertheless, as long as no clear statement can be made, an mTOR based immunosuppressive regimen may be considered in patients after LT for HCC without contraindications.

## 5. Conclusions

The main goal of treatment in HCC patients waiting for LT should be reaching CR as this achievement is crucial in reducing HCC recurrence rates after LT. The applied strategy, number, and combination of treatments were according to our data insignificant. Therefore, selection of treatment modalities should primarily be adjusted in accordance with both patient characteristics such as liver function and tumor properties such as diameter and extension. Microvascular invasion is another major risk factor for HCC recurrence that surely has prognostic relevance. However, this risk factor cannot be modified prior to LT.

## Figures and Tables

**Table 1:** Characteristics of recipients, tumors, and donors.

**Number of subjects**	82

**Characteristics of recipients**	

Age at LT [years]	57.2 ± 9.4
Males	68 (82.9 %)
Body Mass Index [kg/m^2^]	27.56 ± 5.07

**Primary liver disease **	

Alcoholic cirrhosis	21 (25.6 %)
Autoimmune hepatitis	2 (2.4 %)
Hepatitis B	21 (25.6%)
Hepatitis C	21 (25.6 %)
Non-alcoholic fatty liver disease	3 (3.7 %)
Hemochromatosis	2 (2.4 %)
Cryptogenic cirrhosis	8 (9.8 %)
Secondary sclerosing cholangitis	1 (1.2 %)
Alpha-1-deficiency	1 (1.2 %)
Drug-induced liver injury	2 (2.4 %)

**Fibrosis (according to Batts and Ludwig score)**	

none	5 (6.1%)
grade 3	4 (4.9%)
grade 4	73 (89.0 %)

**Time from HCC diagnosis to LT [months]**	7 (IQR 2-12)

**Immunosuppression** **∗**	

Ciclosporin	10 (12.2 %)
Tacrolimus	56 (68.3%)
Everolimus	23 (28.0 %)
Sirolimus	14 (17.1 %)
Mycophenolate Mofetil	62 (75.6 %)

**Survival after LT [months] (all patients)**	49.50 (IQR 24.50-84.75)

male patients	42.00 (IQR 23.50-91.50)
female patients	59.50 (IQR 27.50-74.25)

**Death **	36 (43,9 %)

**Recurrence**	28 (34.1 %)

**Recurrence-free survival [months]**	12.50 (IQR 6-28.25)

**Therapy for HCC (bridging/downstaging) before LT** **∗**	

No therapy	17 (20.7%)
TACE	45 (54.9 %)
SIRT/radiation	8 (9.8%)
Resection	16 (19.5%)
Radiofrequency ablation	8 (9.8%)

**Remission**	

partial	43 (52.4 %)
complete	16 (19.5 %)

**Targeted biopsy before LT**	52 (65.0 %)

**Tumor characteristics**	

AFP pre-LT [ng/ml]	13850 (IQR 5350-135975)
Milan Criteria fulfilled	38 (46.3 %)
Number of HCC lesions:	
1	32 (39.0 %)
2-3	25 (30.5 %)
> 3	9 (11.0 %)
Disseminated tumor infiltration	16 (19.5 %)
Maximum size of HCC lesion(s) [cm]	
< 3	30 (36.6 %)
3-5	25 (30.5 %)
>5	26 (31.7 %)
Microvascular invasion	21 (25.6 %)
Lymphatic invasion	5 (6.1 %)
Tumor Grading	
Complete remission and no biopsy prior to LT	6 (7.3 %)
grade 1	12 (14.6 %)
grade 2	53 (64.6 %)
grade 3	9 (11.0 %)
grade 4	2 (2.4 %)

**Donor Characteristics**	

Age [years]	50.9 ± 15.4
Male sex	52 (63.4 %)
Body Mass Index [kg/m^2^]	26.47 ± 4.33

*∗* More than one option is possible per patient.

Data are presented as mean and standard deviation or median and interquartile range as appropriate.

**Table 2:** Results of univariable and multivariable analyses.

**Variable**	**Univariable analysis**	**Multivariable analysis**		
	**P**	**P**	**OR**	**95**%** CI**
Complete remission before LT	0.010	0.029	0.426	(0.198-0.918)
Vascular invasion	<0.001	0.004	11.357	(2.142-60.199)
Tumor grading	0.030	0.115	-	-
Tumor diameter < 3 cm	0.002	0.040	0.140	(0.022-0.914)
Recipient sex	0.040	0.091	-	-
Number of lesions	0.002	0.149	-	-
Milan criteria	0.007	0.062	-	-
More than one lesion	0.020	0.762	-	-

95% CI, 95% confidence interval; OR, odds ratio; LT, liver transplant.

## Data Availability

Data used to support the findings of this study are included within the article.
